# Description and molecular characterisation of *Babesia ailuropodae* n. sp., a new piroplasmid species infecting giant pandas

**DOI:** 10.1186/s13071-024-06402-6

**Published:** 2024-07-20

**Authors:** Lang Xiong, Guangyou Yang

**Affiliations:** Sichuan Agricultural University, Sichuan, China

**Keywords:** *Ailuropoda melanoleuca*, *Babesia ailuropodae* n. sp, Novel species, Morphology, Phylogeny, Mitochondrial genome

## Abstract

**Background:**

*Babesia* spp. are protozoan parasites that infect the red blood cells of domesticated animals, wildlife and humans. A few cases of giant pandas (a flagship species in terms of wildlife conservation) infected with a putative novel *Babesia* sp. have been reported. However, comprehensive research on the morphological and molecular taxonomic classification of this novel *Babesia* sp. is still lacking. This study was designed to close this gap and formally describe this new *Babesia* sp. infecting giant pandas.

**Methods:**

Detailed morphological, molecular and phylogenetic analyses were conducted to characterise this *Babesia* sp. and to assess its systematic relationships with other *Babesia* spp. Blood samples from giant pandas infected with *Babesia* were subjected to microscopic examination. The 18S ribosomal RNA (18S rRNA), cytochrome b (*cytb*) and mitochondrial genome (mitogenome) of the new *Babesia* sp. were amplified, sequenced and assembled using DNA purified from blood samples taken from infected giant pandas. Based on the newly generated 18S rRNA, *cytb* and mitogenome sequences, phylogenetic trees were constructed.

**Results:**

Morphologically, the *Babesia* sp. from giant pandas exhibited various forms, including round to oval ring-shaped morphologies, resembling those found in other small canine *Babesia* spp. and displaying typical tetrads. Phylogenetic analyses with the 18S rRNA, *cytb* and mitogenome sequences revealed that the new *Babesia* sp. forms a monophyletic group, with a close phylogenetic relationship with the *Babesia* spp. that infect bears (Ursidae), raccoons (Procyonidae) and canids (Canidae). Notably, the mitogenome structure consisted of six ribosomal large subunit-coding genes (LSU1-6) and three protein-coding genes (*cytb*, *cox3* and *cox1*) arranged linearly.

**Conclusions:**

Based on coupled morphological and genetic analyses, we describe a novel species of the genus *Babesia*, namely, *Babesia ailuropodae* n. sp., which infects giant pandas.

**Graphical Abstract:**

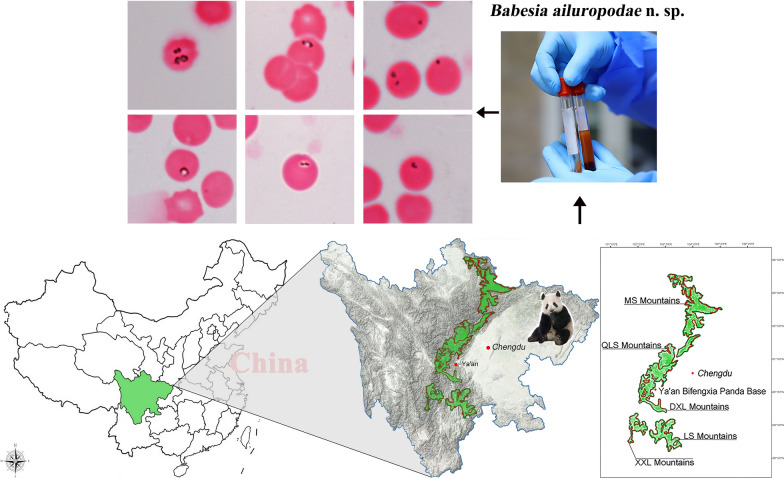

**Supplementary Information:**

The online version contains supplementary material available at 10.1186/s13071-024-06402-6.

## Background

A flagship species in terms of wildlife protection, giant pandas (*Ailuropoda melanoleuca*) are primarily distributed across the Daxiangling, Xiaoxiangling, Qinling, Qionglai and Minshan mountain ranges in the Sichuan, Shaanxi and Gansu provinces of China. Habitat reduction, low reproductive rates and outbreaks of various diseases continue to threaten the survival of giant pandas [1, 2]. Among the threats to giant pandas, diseases caused by parasites are considered one of the significant factors affecting the health of giant pandas [3, 4].

The genus *Babesia* comprises protozoan parasites that are transmitted by ticks; these parasites parasitise red blood cells in livestock, wild animals and humans [5, 6]. There are more than 120 *Babesia* species globally; these species can infect various hosts and induce babesiosis. Haemolysis, fever, jaundice, anaemia and haemoglobinuria are typical clinical signs of babesiosis [7]. Babesiosis primarily affects mammals, including, but not limited to, members of the Canidae, Felidae, Ursidae, Procyonidae, Bovidae, Equidae and Cervidae families. The most common agents of babesiosis include *Babesia canis*, *Babesia rossi*, *Babesia vogeli*, *Babesia conradae*, *Babesia gibsoni*, *Babesia vulpes*, *Babesia felis*, *Babesia cati*, *Babesia lengau*, *Babesia leo*, *Babesia microti*, *Babesia bovis*, *Babesia bigemina*, *Babesia caballi* and a series of newly identified *Babesia* spp. [8–15]. Notably, giant pandas, classified as carnivores within the Ursidae family [16], have exhibited cases of *Babesia* infection, especially among those that have been released into the wild in recent years. These cases have exhibited symptoms, including anaemia, jaundice and haemoglobinuria. Additionally, two cases of naturally occurring *Babesia* infection in wild giant pandas have been documented [7]. Blood protozoa testing was subsequently conducted on all captive giant pandas at the giant panda base in Sichuan Province, as well as on wild giant pandas rescued from the wilderness. The results showed that 14 giant pandas tested positive for *Babesia*, with two wild giant pandas exhibiting typical clinical symptoms of babesiosis, and up to ten parasites were detected within a single erythrocyte, while the remaining positive giant pandas showed no clinical symptoms.

Considering the scarcity of previous research, this study explores the morphological features, phylogenetic relationships and taxonomic status of *Babesia* in giant pandas. This study aims to gain in-depth insights into the characterisation of this parasite within the *Babesia* genus and to offer guidance for future disease prevention and control efforts related to babesiosis.

## Methods

### Samples

**Fig. 1:**
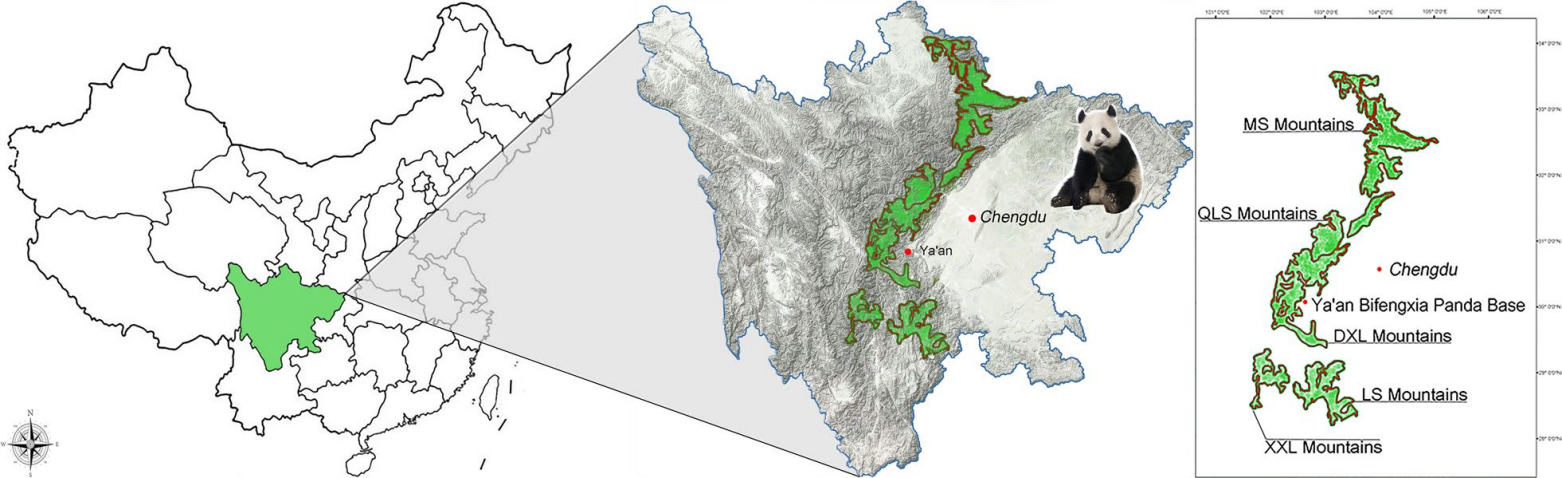
Map showing the distribution of *Babesia*-positive samples collected from giant pandas. *XXL* Xiaoxiangling, *DXL* Daxiangling, *LS* Liangshan, *QLS* Qionglaishan, *MS* Minshan

This study investigated a total of 14 *Babesia*-positive giant pandas—eight from wild giant pandas across China (three from the Qionglai mountain range, three from the Xiaoxiangling mountain range, one from the Liangshan mountain range and one from the Minshan mountain range) and six from captive giant pandas in Sichuan Province (Fig. 1).

### Morphological characterisation of the parasites

Blood smears were made using fresh whole blood collected from giant pandas and were subsequently stained with a Diff-Quick stain kit (Diff-Quick Stain Kit, Solarbio, Beijing, China). The stained blood smears were observed, and photographs were taken using a Zeiss Axio Imager M2 optical microscope (Carl Zeiss, Oberkochen, Germany) with an oil immersion magnification of 1000×; these images were processed using the Zeiss ZEN 2.3 lite software package (Carl Zeiss, Oberkochen, Germany).

### Cloning and sequencing analysis of the mitogenome and 18S rRNA

Following the instructions provided in the DNeasy^®^ Blood and Tissue Kit manual (QIAGEN, Hilden, Germany), DNA extraction was carried out on 200 µl of EDTA-anticoagulated whole blood samples from giant pandas. A NanoDrop spectrophotometer (Nanodrop Technologies, Wilmington, DE) was used to measure the DNA concentrations after the extracted DNA samples were eluted using 200 µl of elution buffer. The isolated DNA was then preserved at –80 °C for use in subsequent molecular biology investigations. Both the 18S ribosomal RNA (18S rRNA) and the mitogenome were amplified using primers designed for the genus *Babesia* (Table 1).

**Table 1:** Sequences of the PCR primers used to amplify the 18S rRNA and mitogenome from giant panda DNA samples

Gene	Primer	Sequence (5′–3′)	Reference
18S rRNA	18SF	TCC TGC CAG TAG TCA TA	[7]
	18SR	TTG TTA CGA CTT CTC CT	
	BP18SF	TCCTGCCAGTAGTCATA	This study
	BP18SR	TTGTTACGACTTCTCCT	
mitogenome	Bab-Forc1	ATWGGATTYTATATGAGTAT	[19]
	Bab-Rev1	ATAATCWGGWATYCTCCTTGG	
	F5F	CTACTACACCCAATAATACAAAAGG	[17]
	F5R	CCATACTGTAGGTATTAATCTATC	
	MTF1-F	GGAAGTGGWACWGGWTGGAC	[18]
	MTF1-R	ACTTTGAACACACTGCTCG	
	MTF2-F	AGGCATGCAATACCGAACAGG	
	MTF2-R	AAGGTACGCCRGGGATAACAGG	
	MTF3-F	AAGGTATGGTGAGACGACATGG	
	MTF3-R	TTCTTAACCCAACTCACGTACC	
	*cox1*-F	GGAAGTGGWACWGGWTGGAC	
	*cox1*-R	TTCGGTATTGCATGCCTTG	
	*cox3*-F	ACTGTCAGCTAAAACGTATC	
	*cox3*-R	ACAGGATTAGATACCCTGG	
	*cytb*-F	CGGTTAATCTTTCCTATTCCTTACG	
	*cytb*-R	TTAGTGAAGGAACTTGACAGGT	
	MCF1	CAGCATGGGATTATAAAACAGT	[20]
	MCR1	GTGGAGACAATAGAGAAGTCG	
	MGF1	CTGTTGCTCCCCAATAACTC	
	MGR1	TTCTTAACCCAACTCACGTACC	

The polymerase chain reaction (PCR) amplification conditions were as follows: initial denaturation at 98 °C for 3 min, 20 s of denaturation at 98 °C, 20 s of annealing at 50–60 °C (depending on the primers used), 10 s of extension at 72 °C, 38 cycles of repetition and a final 5–8 min extension at 72 °C (depending on amplicon size, 1 min/kb). The amplification of the 18S rRNA was accomplished using the primers 18SF/R and BP18SF/R as previously described [7]. The *cytb* gene and the mitogenome were amplified using the primers Bab-Forc1/Rev1, F5F/R, MTF1-F/R, MTF2-F/R, MTF3-F/R, MCF1/R1, MGF1/R1, *cox1*-F/R, *cox3*-F/R and *cytb*-F/R as previously described [17–20].

The PCR amplicons were cloned into the pMD-19T vector (Takara, China) and transformed into *Escherichia coli* DH5α cells. Plasmid DNA was isolated and sent for sequencing by Sangon Biotech (Shanghai, China). Thereafter, the sequencing results were confirmed through sequence alignment using the Basic Local Alignment Search Tool (BLAST) on the National Center for Biotechnology Information (NCBI) website (https://www.ncbi.nlm.nih.gov/BLAST).

### Gene annotation and sequence analysis

Referring to previously published data on *B. canis* (GenBank KC207822), *B. rossi* (KC207823), *B. vogeli* (KC207825) and *B. gibsoni* (KP666169), the mitogenome sequences obtained from the giant panda samples examined in this study were assembled and annotated using the online websites GeSeq (https://chlorobox.mpimp-golm.mpg.de/geseq) and Artemis (https://www.sanger.ac.uk/resources/software/artemis/). Previous annotations of *B. canis*, *B. rossi*, *B. vogeli* and *B. gibsoni* were used to infer the protein-coding genes of the new *Babesia* sp. Sequence data alignment and analyses were conducted using the online websites ORF Finder (https://www.ncbi.nlm.nih.gov/orffinder/) and MAFFT 7.0 (https://mafft.cbrc.jp/alignment/server/). The rRNA genes were further confirmed by referencing the rRNA sequences of *B. gibsoni* (KP666169), *B. rossi* (KC207823) and *B. canis* (KC207822). Moreover, the prediction of transfer RNA (tRNA) genes was performed using tRNAscan-SE 2.0 (http://lowelab.ucsc.edu/tRNAscan-SE/index.html).

### Phylogenetic analysis

Molecular Evolutionary Genetics Analysis (MEGA 5.0) was used to compare the amplified sequences of 18S rRNA, cytochrome b (*cytb*) and the complete mitogenome. The amplified sequences were aligned with those of other similar species listed in GenBank to construct a phylogenetic tree. During the sequence alignment stage, the ClustalW tool was used. The construction of the phylogenetic tree involved various analysis methods, including the maximum likelihood (ML) and neighbour-joining (NJ) methods. The ML tree was constructed using the general time reversible model (G + I) and calculated using the Akaike information criterion (AIC). The NJ tree was constructed with the Tamura-Nei model (G + I) according to the AIC [21, 22]. In the process of constructing the phylogenetic tree, 1000 bootstrap replicates were performed to assess the tree’s reliability [23]. DNAStar was utilised for pairwise genetic analysis of the 18S rRNA and *cytb* datasets, aimed at identifying possible evolutionary differences at the nucleotide level [22]. The first evolutionary tree was constructed based on 61 (almost) full-length sequences of the giant panda *Babesia* 18S rRNA and sequences of protozoa collected from other related species. Additionally, *Toxoplasma gondii* 18S rRNA sequences were used as outgroups. For the analysis of the *cytb* gene, 36 sequences from related protozoan species were used, with the *cytb* gene sequences of *Hepatozoon canis* being used as outgroups. Moreover, in constructing the mitogenome tree, 17 related *Babesia* spp. sequences were used, with *Cytauxzoon felis* sequences serving as outgroups.

## Results

**Family Babesiidae Poche, 1913**

**Genus** ***Babesia*** **Starcovici, 1893**

**Babesia ailuropodae n. sp.**

***Type host:*** giant panda *Ailuropoda melanoleuca* (Mammalia: Ursidae).

***Type locality:*** the mountains of Minshan (31°04′18′′–33°58′28″N, 103°08′24″–105°35′22″E), Qionglaishan (29°38′24″–31°30′36″N, 102°10′48″–103°32′24″E), Daxiangling (29°22′48″–29°48′00″N, 102°36′00″–103°11′24″E), Xiaoxiangling (28°24′36″–29°20′24″N, 101°51′00″–102°33′00″E) and Liangshan (28°12′00″–29°11′24″N, 102°37′12″–103°45′00″E), Sichuan Province, China.

***Other localities:*** unknown.

**Fig. 2:**
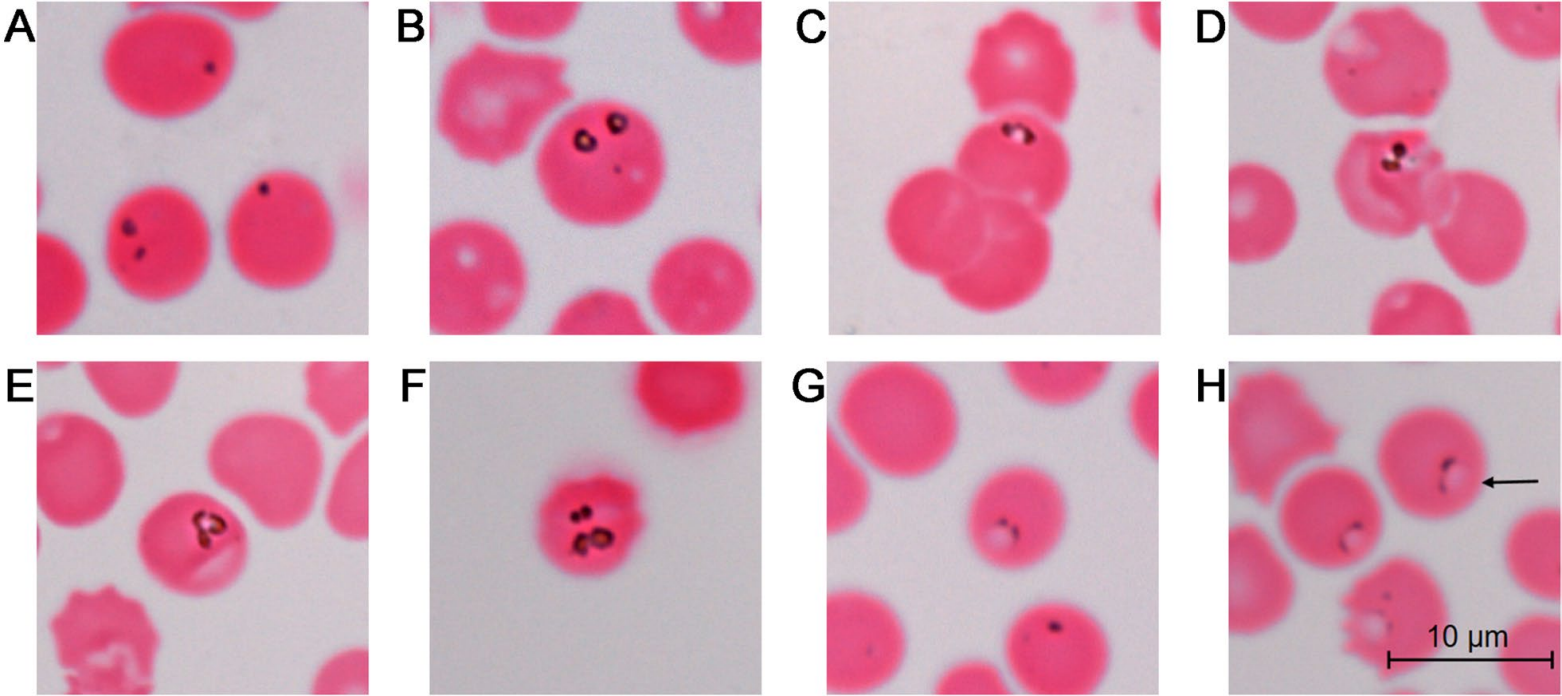
Various morphologies of giant panda *Babesia* merozoites inside erythrocytes, including round-to-oval ring-shaped (**A**, **B**), paired pyriform (**C**, **D**), irregularly shaped (**E**) and tetrad-shaped (**F**) merozoites. *Babesia ailuropodae* n. sp., type material in blood smears from a giant panda (**G**, **H**). The holotype is marked with an arrow in **H**. Diff-Quick stains (modified Wright’s and quick Romanowsky staining). Scale bar, 10 μm

***Type material:*** the study samples, including whole blood, DNA (wild positive sample accession number: GYYXL 2301–8; captive positive sample accession number: GYYXL 2309–14) and dyed thin blood smears (additional file 1: Fig. S1, accession number: GYYXL 230501) from *Babesia*-infected giant pandas containing the holotype (Fig. 2H) were deposited at the Department of Parasitology at Sichuan Agricultural University in Sichuan, China.

***Vector:*** unknown. *Haemaphysalis flava* Neumann, 1897 is suspected [24, 25].

***Representative DNA sequences:*** GenBank accession numbers PP117080-PP117093 for 18S rRNA, PP215402-PP215412 for *cytb* and PP236906 for the mitogenome.

***ZooBank registration:*** details of the new *Babesia* species were submitted to ZooBank in accordance with the guidelines mentioned in Article 8.5 of the 2012 amendment to the International Code of Zoological Nomenclature (ICZN) [26]. The Life Science Identifier (LSID) of the article is urn:lsid:zoobank.org:pub:5BD943D8-989D-4DB2-8373-86F7673E3686. The LSID for the new *Babesia ailuropodae* n. sp. is urn:lsid:zoobank.org:act:55427CD6-EF60-4981-B679-886FEAC7A5B4.

***Etymology:*** the species name was derived from the genus name of the type host.

### Morphological description

In the stained blood smears, round to oval ring-shaped, paired pyriform, irregularly shaped and tetrad-shaped forms were observed (Fig. 2). There were varying numbers of parasites infecting erythrocytes, with individual ring-shaped parasites constituting the majority. Round-to-oval-shaped merozoites exist as single or double parasites within infected erythrocytes. The range of parasites infecting a single erythrocyte was between 1 and 10. The following were the size ranges of the different parasitic forms: round-to-oval ring-shaped merozoites measuring 0.926–2.505 µm (mean ± standard deviation: 1.574 ± 0.439) in length and 0.665–2.29 µm (1.365 ± 0.395) in width (*n* = 30); paired pyriform structures measuring 1.987–2.686 µm (2.350 ± 0.219) in length and 1.072–1.763 µm (1.399 ± 0.236) in width (*n* = 11); irregularly shaped structures measuring 1.756–3.917 µm (2.381 ± 0.849) in length and 1.204–3.331 µm (1.982 ± 0.705) in width (*n* = 8); and tetrads measuring 2.815–4.074 µm (3.519 ± 0.467) in length and 2.022–3.198 µm (2.605 ± 0.358) in width (*n* = 12). Moreover, the average blood parasitemia in the erythrocytes of the affected animals was 44.60 ± 5.36% (positive cells, 70.143 ± 8.030; total cells, 160.429 ± 14.932).

### Sequence analysis of the 18S rRNA and *cytb*

We successfully cloned nearly complete (1679 bp) 18S rRNA sequences from all 14 positive giant panda blood samples. Compared with the previously reported sequence of the *Babesia* sp. EBP strain in giant pandas (MT256300), the new *Babesia ailuropodae* n. sp. cloned sequence exhibited an additional 75 base pairs while maintaining a high similarity (99.75%). Furthermore, the 18S rRNA consensus sequence (PP117082) of *Babesia ailuropodae* n. sp. showed significant similarities with those of Japanese black bears (99.51%, AB586027) and brown bears (99.49%, AB566229). Simultaneously, the similarity to *Babesia* spp. from canids (AY190123, KR017880) and raccoons (OK524313) exceeded 99%. Compared with *B. felis*, which infects felids (AY452707) in other regions, the similarity reached only 86.52% (Table 2).

**Table 2:** Comparison of the identities between the complete 18S rRNA sequence (1679 bp) of the giant panda *Babesia* and other published *Babesia* sequences

Species	Host	Length (bp)	Cover (%)	Identity (%)	Accession no.
*Babesia* sp*.* EBP	Giant panda	1604	95	99.75	MT256300
*Babesia* sp*.* Iwate	Black bear	1635	96	99.51	AB586027
*Babesia* sp*.* EBB	Brown bear	1568	93	99.49	AB566229
*Babesia* sp*.* Akita	Domestic dog	1678	99	99.40	AY190123
*B. gibsoni*	Red panda	1721	100	99.23	OK524313
*Babesia* sp*.* maned wolf	Maned wolf	1652	98	99.09	KR017880
*Babesia* sp*.* AJB-2006	Raccoon	1618	96	98.95	DQ028958
*Babesia* sp*.* FP44	Florida panther	1734	100	98.16	DQ329138
*Babesia* sp*.* venatorum	Human	1727	100	97.69	KF724377
*B. capreoli*	Horse	1724	100	97.45	KX839234
*B. odocoilei*	*Odocoileus virginianus*	1723	100	97.57	U16369
*B. divergens*	Reindeer	1724	100	97.39	AY098643
*B. gibsoni*	Domestic dog	1719	100	96.09	HG328235
*B. canis*	Domestic dog	1711	100	93.70	L19079
*B. major*	Domestic cow	1684	100	92.40	EU622907
*B. lengau*	Domestic cat	1648	98	88.54	KC790443
*B. leo*	Lion	1690	100	87.24	AF244911
*B. felis*	Domestic cat	1627	97	86.52	AY452707

Eleven *cytb* gene sequences (1092 bp) from positive panda blood samples were successfully cloned. BLASTn analysis demonstrated that the *cytb* gene consensus sequence (PP215403) from *Babesia ailuropodae* n. sp. shared 82.6% similarity with that of *B. vogeli* (KC207825). Its similarity with the *cytb* gene sequences of *B. bigemina* (AB499085), *B. bovis* (EU075182), *B. ovate* (LC146481) and *B. naoakii* (LC684769) was less than 80% (Table 3).

**Table 3:** Comparison of the identities between the complete *cytb* sequence (1092 bp) of the giant panda *Babesia* and other published *Babesia* sequences

Species	Length (bp)	Identity (%)	Accession
*B. vogeli*	1092 bp	82.60	KC207825
*Babesia* sp*.* Xinjiang	1092 bp	82.26	MK962313
*B. rossi*	1092 bp	82.23	KC207823
*B. gibsoni*	1092 bp	82.14	KP666169
*B. caballi*	1092 bp	81.88	AB499086
*Babesia* sp. pudui	1085 bp	81.51	ON995403
*Babesia* sp. Coco	1092 bp	81.35	KC207824
*B. motasi*	1092 bp	80.00	MN605890
*B. bigemina*	1092 bp	79.45	AB499085
*B. bovis*	1092 bp	78.75	EU075182
*B. ovata*	1092 bp	78.81	LC146481
*B. naoakii*	1092 bp	77.92	LC684769

We found that *Babesia* gene sequences from giant pandas collected from different geographical sources exhibited relatively conserved intraspecific genetic relationships, with 14 high-quality 18S rRNA sequences showing 99.4–100% similarity and nine high-quality *cytb* sequences displaying 97.4–100% nucleotide identity.

### Phylogenetic analysis

**Fig. 3:**
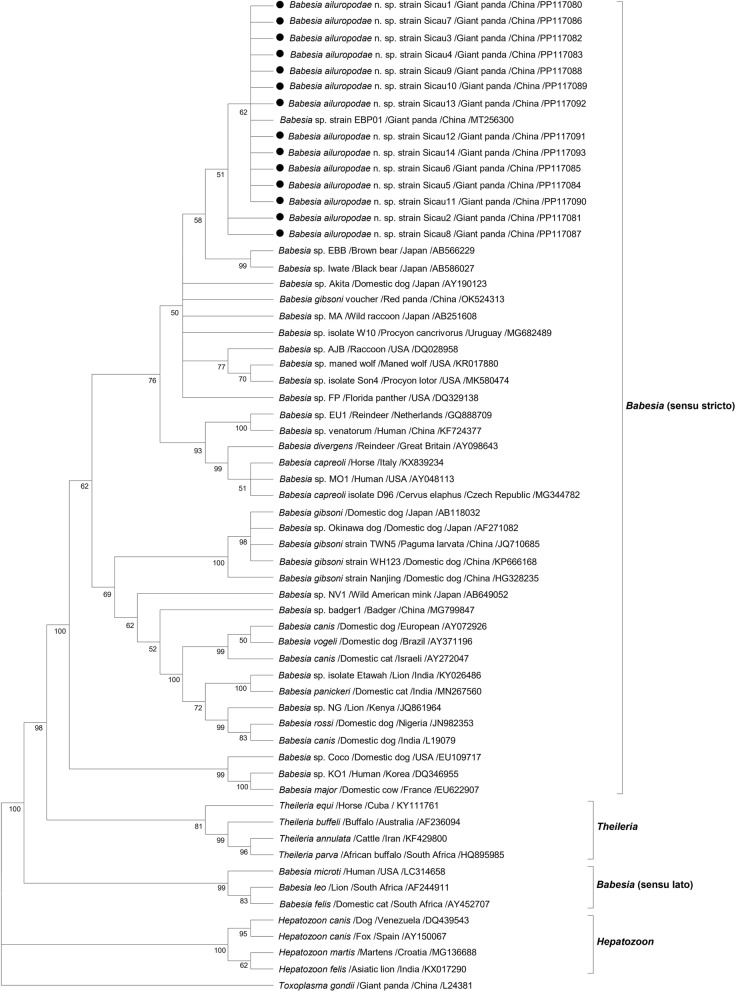
Phylogenetic analysis of the nearly complete giant panda *Babesia* 18S rRNA sequences was conducted using the maximum likelihood method. The 18S rRNA sequence of *T. gondii* served as the outgroup. With a sequence length of 1679 bp, the general time reversible (G + I) model was used to construct the ML tree. The analysis did not include gaps or missing data. For each sequence, information on the host, the country of origin and the GenBank accession number are provided. Only bootstraps > 50% are shown, and the bootstrap values are based on 1000 repetitions

Phylogenetic analysis of *Babesia*, encompassing 61 sequences, revealed four distinct clades: *Babesia* (senso stricto), *Babesia* (sensu lato), Theileriidae, and Hepatozoidae. The 18S rRNA sequences from the 14 positive giant panda blood samples in this study clustered with a published *Babesia* sequence from giant pandas (MT256300) within the *Babesia* (s.s.) clade. Specifically, the giant panda *Babesia* 18S rRNA sequence showed highest similarity to those of Japanese black bears (AB586027) and brown bears (AB566229), with decreasing similarity to that of the *Babesia* sequences from Japanese domestic dogs (AY190123), Chinese red pandas (OK524313) and Japanese wild raccoons (AB251608). These sequences also exhibited similarity to *Babesia* strains infecting other canids and procyonids. The remaining branches included the *Babesia* (s.l.) clade (three sequences), Theileriidae clade (four sequences) and Hepatozoidae clade (four sequences), positioned further apart from the giant panda *Babesia* sequences, which included species such as *B. leo* (AF244911), *B. felis* (Y452707), *T. equi* (KY111761), *T. parva* (HQ895985), *H. canis* (AY150067), and *H. felis* (KX017290) (Fig. 3).

**Fig. 4:**
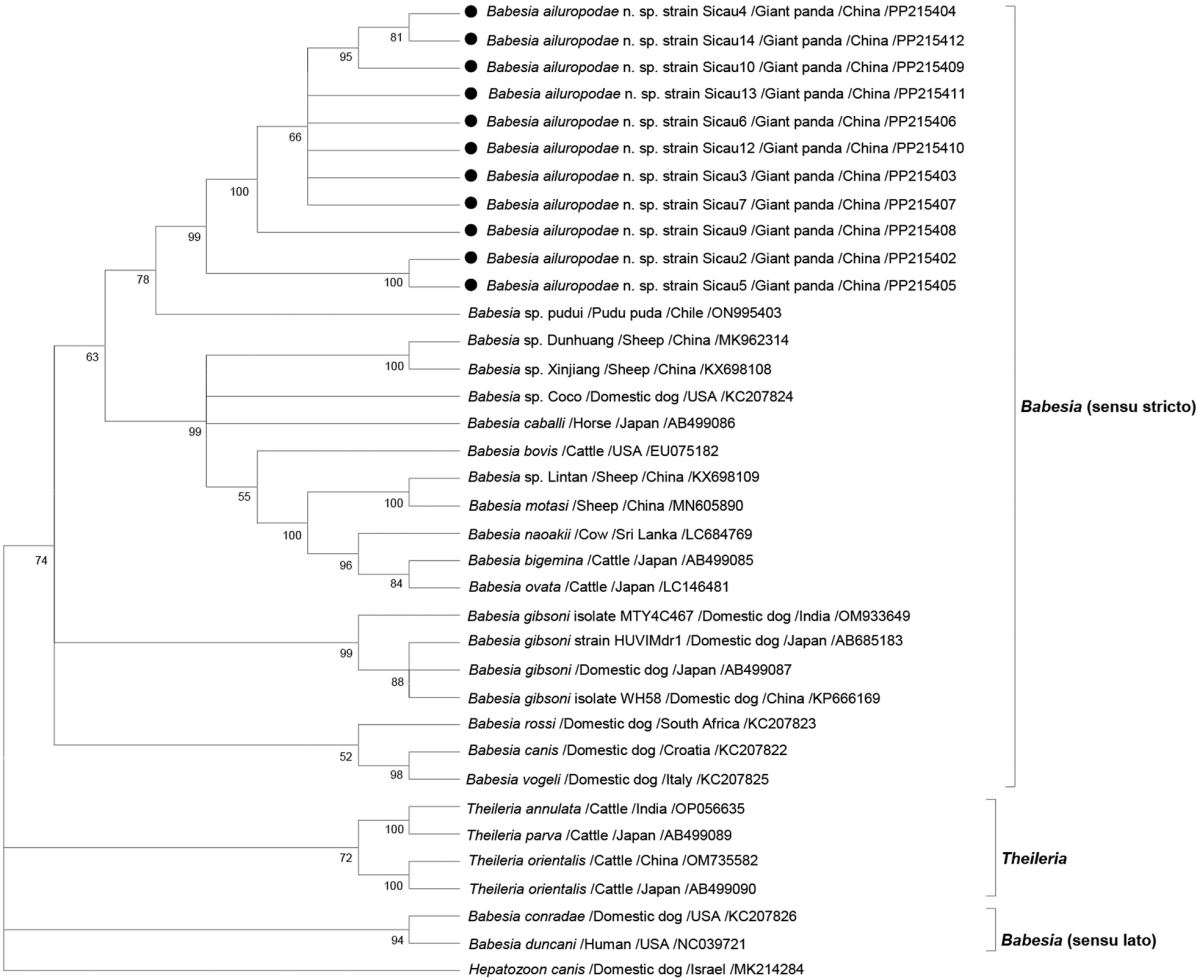
Phylogenetic analysis of the giant panda *Babesia cytb* gene sequences was conducted using the maximum likelihood method. The *cytb* sequence of *H. canis* served as the outgroup. With a sequence length of 1092 bp, the general time reversible (G + I) model was used to construct the ML tree. The analysis did not include gaps or missing data. For each sequence, information on the host, the country of origin and the GenBank accession number are provided. Only bootstraps > 50% are shown, and the bootstrap values are based on 1000 repetitions

Furthermore, the *cytb* gene sequences of 11 positive giant panda blood samples were aligned with 25 protozoan *cytb* gene sequences obtained from the GenBank database to construct an evolutionary tree, with the *H. canis cytb* sequence serving as the outgroup. The evolutionary tree revealed three main clades; one clade represented the *Babesia* (s.s.) group, where all 11 *cytb* sequences of the giant panda *Babesia* formed a distinct clade and were situated within this group. They shared a close phylogenetic relationship with other *Babesia cytb* sequences, primarily including *B*. sp. pudui, *B*. sp. Dunhuang, *B*. sp. Coco, *B. rossi*, *B. gibsoni*, *B. canis* and some *Babesia* subspecies. Additionally, the other two main clades included *Babesia* (s.l.) and Theileriidae, which were relatively distantly related to the *cytb* clade of the giant panda *Babesia* and were situated closer to the outgroup (Fig. 4).

**Fig. 5:**
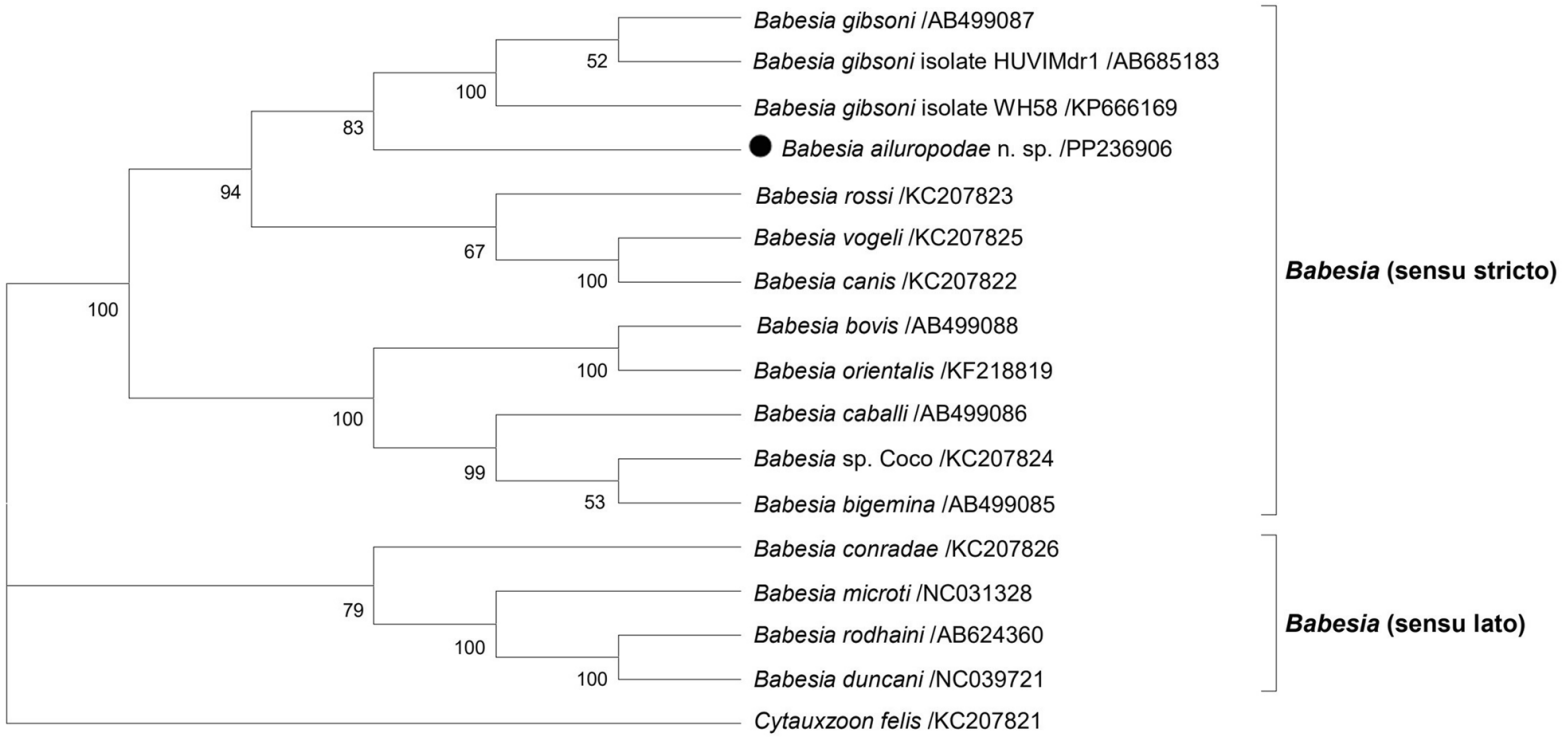
Phylogenetic analysis of the giant panda *Babesia* mitogenome was conducted using the maximum likelihood method. The sequence of *C. felis* served as the outgroup. With a sequence length of 5609 bp, the TN93 + G model was used to construct the NJ tree. The analysis did not include gaps or missing data. Only bootstraps > 50% are shown, and the bootstrap values are based on 1000 repetitions

The mitogenome tree showed two primary clades: one representing the *Babesia* (s.s.) group, which was distinctively separate from the outgroup *C. felis*. Within *Babesia* (s.s.), the mitogenome sequence of the giant panda *Babesia* was closely related to that of *B. gibsoni*, *B. rossi* and *B. canis*. The remaining clades belonged to the *Babesia* (s.l.) group, positioned nearer to the outgroup *C. felis*, and primarily included *B. conradae*, *B. microti*, *B. rodhaini* and *B. duncani* (Fig. 5).

### Mitogenome

**Fig. 6:**
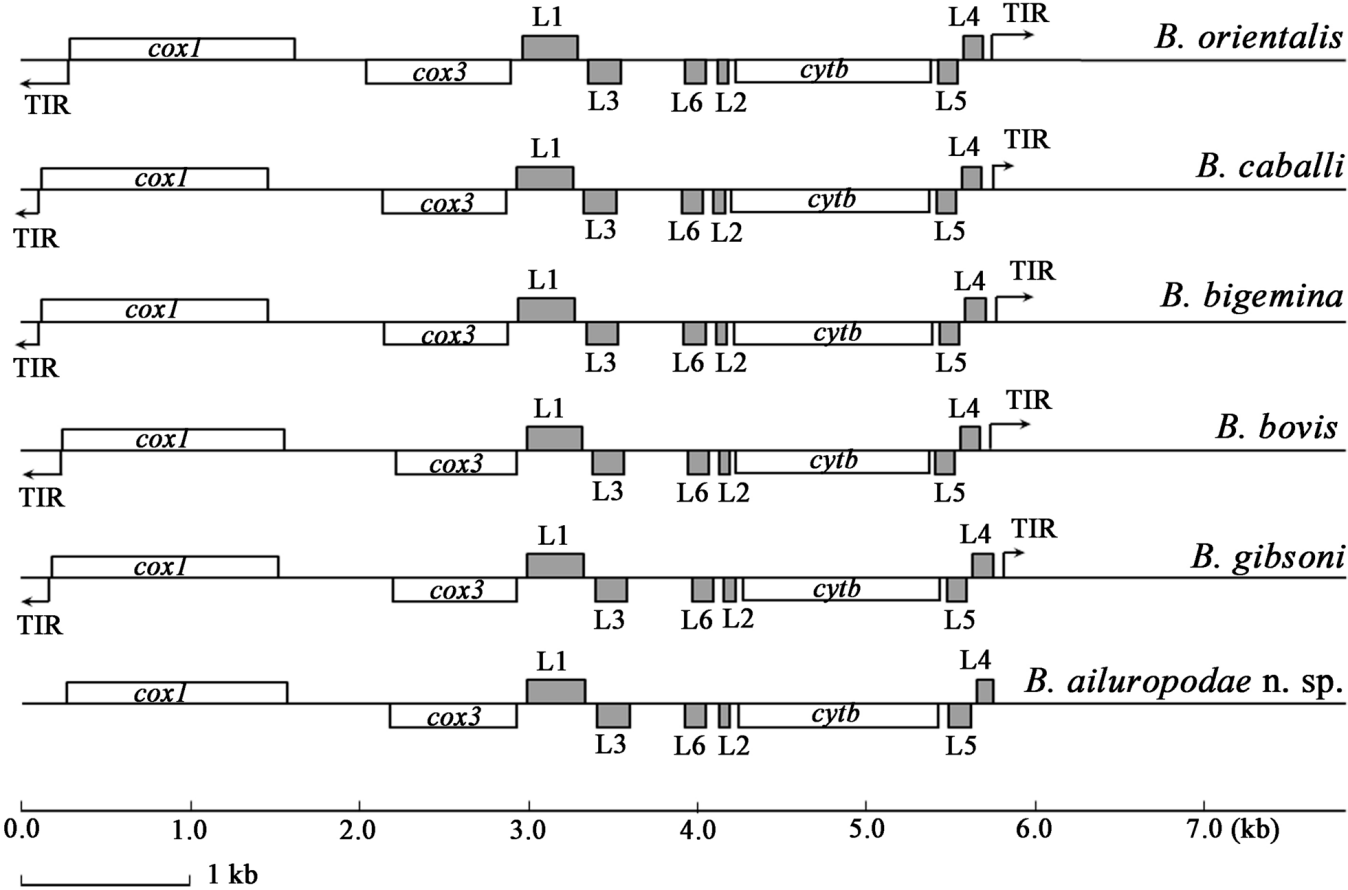
Comparison results of the mitogenome map of the giant panda *Babesia* and other published *Babesia* linear mitogenome maps, including *B. gibsoni* (AB499087), *B. bovis* (AB499088), *B. bigemina* (AB499085), *B. caballi* (AB499086) and *B. orientalis* (KF218819). White boxes represent protein-encoding genes (*cox1*, *cox3* and *cytb*). The grey boxes represent large subunits (LSU1-6), and the arrows represent terminal inverted repeats (TIRs)

The giant panda *Babesia* mitogenome, designated with GenBank accession number PP236906, spans 5608 bp and includes six ribosomal large subunit genes (LSU) and three protein-coding genes (*cox1*, *cox3* and *cytb*), with no amplification of TIR at either end. The sizes of the *cox1*, *cox3* and *cytb* genes were 1431 bp, 642 bp and 1092 bp, respectively. The sizes of LSU1, LSU2, LSU3, LSU4, LSU5 and LSU6 were 321 bp, 35 bp, 111 bp, 48 bp, 72 bp and 43 bp, respectively. Unlike other Apicomplexan parasites, it lacks tRNA and exhibits a linear structure typical of *Babesia* mitogenomes, resembling those of *B. gibsoni* (AB499087), *B. bovis* (AB499088), *B. bigemina* (AB499085), *B. caballi* (AB499086) and *B. orientalis* (KF218819) [27–30]. The mitogenome structure and gene arrangement are consistent with this lineage, which typically does not exceed 6000 bp (Fig. 6).

## Discussion

To date, *Babesia* infections have been detected in animals from the Canidae, Felidae, Ursidae, and Procyonidae families worldwide, all of which are closely related to giant pandas. Among canids [31–42], including domestic dogs, wild dogs, foxes, raccoon dogs, black-backed jackals, grey wolves, coyotes and maned wolves, as well as felids such as domestic cats, wild cats, bobcats, caracals, ocelots, cheetahs, leopards, cougars, jaguars and lions, *Babesia* infections have been identified [43–48]. Additionally, *Babesia* infections have been diagnosed in Ursidae animals, including black bears in Japan and the USA, brown bears in Japan, polar bears and sun bears [49–55]. Moreover, reports of *Babesia* infection in Procyonidae animals have also been increasing steadily [56–60]. Recently, confirmed cases of *Babesia* infection have been detected in wild giant pandas undergoing training [7]. This study systematically classifies the *Babesia* species found in giant pandas, identifying it as a new taxonomic unit meeting the criteria for a new species according to the ICZN guidelines [26]. According to ICZN standards, naming a newly discovered parasite involves several steps: providing a valid species description encompassing the morphological, biological and potential taxonomic characteristics; designating a type specimen; and assigning a Latinised name that conforms to international naming conventions to ensure uniqueness. This study meticulously described the shape, size, vector, type locality, type host, type material, ZooBank registration, designation and taxonomic status of *Babesia* in giant pandas. Consequently, the study named this species *Babesia ailuropodae* n. sp. The species name reflects the giant panda (*Ailuropoda melanoleuca*) as the primary natural host of this Apicomplexan parasite [7].

The morphological analysis in this study reveals that *Babesia* in giant pandas exhibits pleomorphism within erythrocytes, typically appearing as individual ring-shaped parasites. This parasite predominantly parasitises the periphery of erythrocytes and shares parasitic characteristics similar to small *Babesia* species found in canids and felids [33, 61–63]. Individual parasites of *Babesia* in giant pandas exhibit a size range of 1.365 µm × 1.574 µm in their various morphological forms. Additionally, *B. ailuropodae* n. sp. also appears in irregular forms, differing somewhat from *Babesia* species found in canids and felids. Furthermore, they share similarities in tetrad morphology with *B. canis*, *B. microti*, *B. duncani*, *B. conradae*, *B. negevi* and *B. panickeri* [64–66]. Compared with the larger *Babesia* species reported in canids, such as *B. canis* (2 µm × 5 μm), *B. vogeli* (2.5 µm × 4.5 μm) and *B. rossi* (2 µm × 5 μm), *B. ailuropodae* n. sp. (1.365 ± 0.395 µm × 1.574 ± 0.439 μm) is relatively smaller. In contrast, compared with smaller *Babesia* species in felids such as *B. lengau* (0.6 µm × 2.3 µm) and *B. leo* (0.62 µm × 1.73 µm), *B. ailuropodae* n. sp. tends to be larger [46, 67]. The size of *B. ailuropodae* n. sp. overlaps significantly with that of *B. gibsoni* (1 µm × 3 μm), placing it in the category of medium-sized parasites [65, 68]. These distinct morphological characteristics indicate that the *Babesia* species found in giant pandas is unique. This finding presents an exciting prospect, as research on *Babesia* in Ursidae and Procyonidae has thus far been limited to epidemiological investigations, with detailed studies on morphology and systematic classification yet to be conducted.

Although a single-gene evolutionary tree may not fully capture the parasite’s position in species evolution, combining multiple gene features with potentially diverse evolutionary histories can construct a more accurate phylogenetic tree that reflects the true relationships among related species [69]. This study conducted a genetic analysis of the 18S rRNA and *cytb* genes in the giant panda *Babesia* to elucidate the evolutionary relationships of the newly identified species *B. ailuropodae* n. sp. The results indicated that *Babesia* isolates from giant pandas form a distinct evolutionary branch in different phylogenetic trees (Figs. 3 and 4), clearly distinguishing them from other *Babesia* species. This finding supports the idea that *B. ailuropodae* n. sp. could be unique to giant pandas because of its distinctive molecular evolutionary history.

In this study, four branches – *Babesia* (s.s.), *Babesia* (s.l.), *Theileria* and *Hepatozoon* – were visible in the phylogenetic tree built using the 18S rRNA gene sequence. Within *Babesia* (s.s.), *B. ailuropodae* n. sp. formed a sister clade along with the *Babesia* gathered from black bears and brown bears in Japan. It shares a high degree of similarity with the *Babesia* collected from domestic dogs in Japan and from the red panda in China [51, 70]. A geographical analysis revealed that the distributions of black bears, brown bears and domestic dogs in Japan do not overlap with those of giant pandas in China, which largely impedes inter-species disease transmission. However, this study revealed significant genetic similarities in the *Babesia* strains infecting these animals. The strong evolutionary kinship among giant pandas, black bears and brown bears, which are all members of the order Carnivora, likely explains this phenomenon. Despite their geographical separation, their genetic similarity suggests the potential for disease transmission among these species [71]. *H. flava*, primarily found in East Asia, including China, Japan and Korea, is suspected to be a vector for giant pandas and has been detected in bear species in Japan, indicating its potential role in disease transmission [24, 25]. Therefore, we speculate that *H. flava* may serve as a common vector for *Babesia* infection. In this study, within the *Babesia* (s.s.) group, *B. ailuropodae* n. sp. and *Babesia* from the red panda were closely related, showing 99.23% sequence similarity in their 18S rRNA (OK524313), despite significant differences in their hosts’ evolutionary lineages. Notably, the red panda, a member of the Ailuridae family within the superfamily Musteloidea, is evolutionarily distinct from the giant panda, which shares closer kinship with members of the bear family (Ursidae) [16, 72, 73]. Despite their differences, giant pandas and red pandas have both inhabited the same geographical region and have undergone convergent evolution over time, thereby adapting similarly to environmental pressures and acquiring morphological and physiological similarities [74]. Instances of *Babesia* infection in Procyonidae animals are well documented worldwide, and the similarity in genetic sequences of the *Babesia* derived from these animals corresponds to the phylogenetic relationships among their hosts [57–59, 75, 76]. Simultaneously, the 18S rRNA sequences of *Babesia* in Japanese domestic dogs and those in giant pandas showed high similarity in this study, possibly due to ticks incidentally infecting Japanese domestic dogs, which may have originated from Japanese wild raccoons. This hypothesis was supported by the evolutionary tree analysis comparing *Babesia* sequences from Japanese domestic dogs with the 18S rRNA sequences from raccoons in Japan and the United States of America [14, 59, 70]. These findings further support that *Babesia* infections in domestic dogs across various regions – Japan (AB118032, AF271082), China (HG328235, KP666168), Brazil (AY371196), India (L19079), Nigeria (JN982353), the USA (EU109717), and Europe (AY072926) – exhibit distinct evolutionary branches compared with *B. ailuropodae* n. sp. within *Babesia* (s.s.). Therefore, based on the 18S rRNA sequence classification analysis, the giant panda *Babesia* demonstrated a close phylogenetic relationship with *Babesia* species found in the bear (Ursidae), panda (Ailuridae), raccoon (Procyonidae) and dog (Canidae) families.

The *cytb* sequences of *B. ailuropodae* n. sp. differed by 1 to 53 base pairs from those of other *Babesia* species. Alignments of 82.6% with canine *Babesia* species and 77.92% with *B. naoakii* were obtained. This variability indicates its genetic independence in evolutionary development. Overall, the *cytb* gene sequences of 11 *B. ailuropodae* n. sp. formed a distinct branch, mirroring the branching pattern observed in the 18S rRNA analysis. This underscores the unique position of the giant panda *Babesia* within *Babesia* (s.s.). The *cytb* sequence of *B. ailuropodae* n. sp. showed close phylogenetic relationships with those of *B.* sp. Pudui (ON995403), *B.* sp. Dunhuang (MK962314), *B.* sp. Coco (KC207824), *B. caballi* (AB499086), *B. gibsoni* (OM933649) and *B. rossi* (KC207823). This genetic evolutionary analysis was consistent with the morphological characteristics of this species, supporting the classification of the giant panda *Babesia* as an independent taxonomic unit. Additionally, *B. conradae* (KC207826) and *B. duncani* (NC039721) within *Babesia* (s.l.) were found to be more distantly related to the outgroup, indicating a less close phylogenetic relationship with *B. ailuropodae* n. sp. This divergence may be attributed to environmental selection pressures between the parasites and their hosts, as well as inherent evolutionary differences among various parasitic species [62].

To date, the mitogenomes of most piroplasmids, including *B. canis*, *B. vogeli*, *B. rossi*, *B. gibsoni*, *B. bovis*, *B. orientalis*, *B. caballi*, *B. bigemina*, *B. conradae*, *B. microti*, *B. rodhaini*, *B. duncani*, *T. equi*, *T. parva*, *T. orientalis*, *H. canis* and *C. felis,*, have been extensively studied and analysed [17, 18, 29, 77–79]. The study of mitogenomes has contributed significantly to the provision of crucial information about the biological characteristics, inheritance and species categorisation of pathogens [27]. In this study, the mitogenome of the giant panda *Babesia* was amplified, sequenced, assembled and then compared with those of other piroplasms from the GenBank database. The findings revealed that the mitogenome of the giant panda *Babesia* shares substantial similarity in size, structure and content with those of other *Babesia* species. It contains three protein-coding genes (*cox1*, *cox3* and *cytb*) and six large subunit rRNA genes (LSU1-6), which are arranged linearly. However, attempts to amplify the two TIRs of the mitogenome using various methods, including high-throughput deep sequencing, inverted PCR, designing specific primers and optimising the PCR conditions, were unsuccessful in this study. This failure may be due to the extensive diversity and variability of TIRs among *Babesia* species within the phylum Apicomplexa, compounded by potential host-specific factors influencing the availability of TIRs in the mitogenomes of the studied *Babesia* species [17, 30, 80]. The genetic analysis in this study revealed that the mitogenome of the giant panda *Babesia* is closely related to those of *B. gibsoni*, *B. rossi*, *B. vogeli* and *B. canis*, which form a sister branch. In contrast, it exhibits a more distant branching relationship with *B. duncani*, *B. rodhaini*, *B. microti* and *B. conradae*.

## Conclusions

Based on morphological and molecular analyses (including mitogenome), we formally described a new species of piroplasmid infecting giant pandas in China, namely, *Babesia ailuropodae* n. sp. Further research is required to confirm the full host range and geographical distribution of the *Babesia ailuropodae* n. sp. vector.

### Supplementary Information

Additional file 1: Fig. S1 Position of the holotype in the slide (accession number: GYYXL 230501).

## Data Availability

The molecular data have been deposited in GenBank under the following accession numbers: 18S rRNA, PP117080-PP117093; *cytb*, PP215402-PP215412; and mitogenome, PP236906.

## Abbreviations

*B. ailuropodae* n. sp.: *Babesia ailuropodae* n. sp.
*T. gondii*: *Toxoplasma gondii*
*H. canis*: *Hepatozoon canis*
*C. felis*: *Cytauxzoon felis*
18S rRNA: 18 Svedberg ribosomal RNA
*cox*: Cytochrome c oxidase
*cytb*: Cytochrome b
Mitochondrial genome: Mitogenome
s.s.: Sensu stricto
s.l.: Sensu lato
PCR: Polymerase chain reaction
LSU: Large subunit
ICZN: International code of zoological nomenclature
