# Supplementary figures and images for: Description and molecular characterisation of Babesia ailuropodae n. sp., a new piroplasmid species infecting giant pandas
Source: Parasit Vectors. 2024 Jul 20;17:315. doi: 10.1186/s13071-024-06402-6 (PMC11265107; doi:10.1186/s13071-024-06402-6)

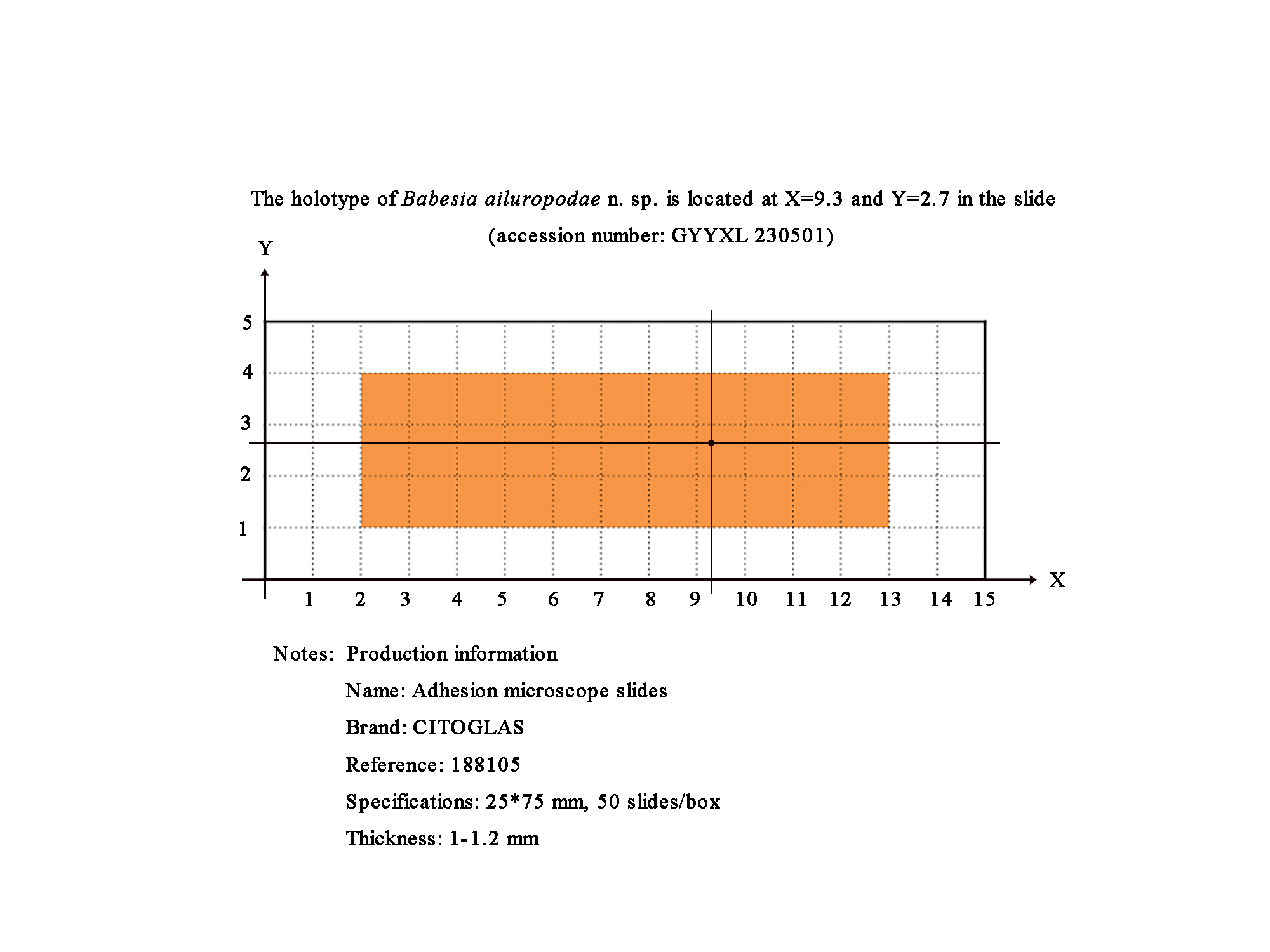

Supplement: Supplementary file 1 — Additional file 1: Fig. S1 Position of the holotype in the slide (accession number: GYYXL 230501). [file 13071_2024_6402_MOESM1_ESM.jpg]
